# Black-blood dynamic contrast-enhanced coronary artery wall MRI: a potential tool for kinetic-modeling-based wall inflammation assessment

**DOI:** 10.1186/1532-429X-15-S1-W13

**Published:** 2013-01-30

**Authors:** Z Fan, J Xie, Y He, Y Natsuaki, N Jin, DS Berman, D Li

**Affiliations:** 1 Cedars-Sinai Medical Center, Los Angeles, CA, USA; 2 Radiology, Beijing Anzhen Hospital, Beijing, China; 3 Siemens Healthcare, Los Angeles, CA, USA; 4 Siemens Healthcare, Columbus, OH, USA

## Background

Dynamic gadolinium contrast-enhanced (DCE) vessel wall MRI has recently been used to compute a set of model-based contrast kinetic parameters (e.g. K^trans^ and V_p_) that can well characterize the extent of inflammation in carotid plaques [1,2]. However, no studies have shown its feasibility in coronary artery wall, presumably due to the technical challenges in imaging such a constantly-moving, ultra-small structure and potential difficulty in visually distinguishing the wall from the hyperintense lumen with conventional DCE techniques. This work aimed to develop a black-blood navigator-gated ECG-triggered T1-weighted sequence for DCE MRI of coronary vessel wall.

## Methods

**Figure 1:**
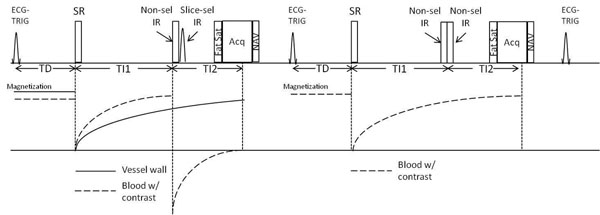
Sequence diagram of the ECG-triggered and navigator-gated SR-DIR prepared RF spoiled GRE sequence. Black-blood and bright-blood images data are collected in an interleaved fashion. Blood signal is suppressed by a combination of SR and dual-IR. SR: saturation recovery; IR: inversion recovery.

An SR-DIR (saturation recovery combined with double inversion recovery) preparation is combined with an RF spoiled GRE sequence to achieve: 1) To create T1-weighting for vessel wall; 2) To consistently null the blood signal with a fixed inversion time combination (TI1 and TI2) (Fig. 1). Bright-blood acquisition is interleaved with black-blood acquisition to enable arterial blood signal measurement as needed in kinetic modeling [3].

Ten healthy volunteers (1 F, 9 M; age 22-45 years) were scanned at 3T using a 6-channel body matrix coil and spine coil. DCE imaging was performed using the developed technique at one single slice selected from one of major coronary arteries. Relevant imaging parameters: resolution = 0.8 × 0.8 × 4.0 mm^3^, TI1/TI2 = 350/40 ms. One-frame pre-contrast scan was followed by repetitive contrast-enhanced scans (1-2 min/frame, > 15 min), along with intravenous contrast (0.2 mmol/kg gadopentetate dimeglumine) injection and saline flush (30 ml) both at 0.2 ml/s. The changes in signal intensity of coronary vessel wall and lumen were obtained from black-blood and bright-blood images, respectively.

## Results

**Figure 2:**
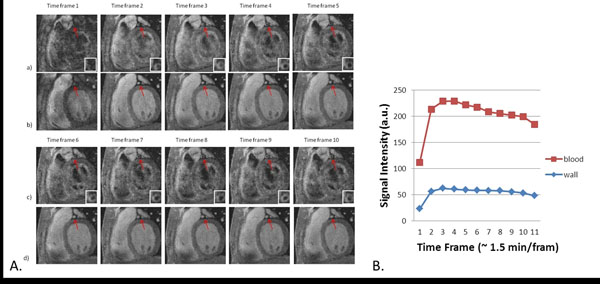
A. Representative dynamic black-blood (a, c) and bright-blood (b, d) images throughout the contrast injection process. B. Typical signal intensity vs. time curves of coronary vessel wall and blood.

The lumen signal was consistently nulled in the black-blood acquisition (Fig. 2A a, c) and vessel wall was clearly differentiated from the lumen. The sharp wash-in and slow wash-out process of the blood signal was observed in all the volunteers (Fig. 2B). From the 10 subjects, K^trans^ = 0.031±0.020 min^-1^, K_ep_ = 0.226±0.105 min^-1^, and V_p_ = 38.37±23.34%. The kinetic model fitted the acquired data well (R2 = 0.80±0.07, ANOVA analysis).

## Conclusions

With SRDIR preparation, black blood imaging can be fulfilled consistently regardless of blood T1 value. This could improve the accuracy of vessel wall signal measurement and make this technique feasible for coronary vessel wall that is much thinner than carotid vessel wall. A feasibility study applying this technique to clinical patients with stable angina is underway.

